# Insufficient sleep and weekend recovery sleep: classification by a metabolomics-based machine learning ensemble

**DOI:** 10.1038/s41598-023-48208-z

**Published:** 2023-11-30

**Authors:** Marie Gombert, Nichole Reisdorph, Sarah J. Morton, Kenneth P. Wright, Christopher M. Depner

**Affiliations:** 1 Department of Pediatrics, Obstetrics and Gynecology, University of Valencia, 46010 Valencia, Spain; 2 Center for Health Sciences, SRI International, Menlo Park, CA USA; 3 Skaggs School of Pharmacy and Pharmaceutical Sciences, University of Colorado Anschutz Medical Campus, Aurora, CO USA; 4 Sleep and Chronobiology Laboratory, Department of Integrative Physiology, University of Colorado Boulder, 1725 Pleasant Street; Clare Small 114, Boulder, CO 80309-0354 USA; 5 Division of Endocrinology, Metabolism, and Diabetes, University of Colorado Anschutz Medical Campus, Aurora, CO 80045 USA; 6 Department of Health and Kinesiology, University of Utah, 250 S 1850 E; HPER North, RM 206, Salt Lake City, UT 84112 USA

**Keywords:** Biochemistry, Metabolomics, Medical research, Biomarkers

## Abstract

Although weekend recovery sleep is common, the physiological responses to weekend recovery sleep are not fully elucidated. Identifying molecular biomarkers that represent adequate versus insufficient sleep could help advance our understanding of weekend recovery sleep. Here, we identified potential molecular biomarkers of insufficient sleep and defined the impact of weekend recovery sleep on these biomarkers using metabolomics in a randomized controlled trial. Healthy adults (n = 34) were randomized into three groups: control (CON: 9-h sleep opportunities); sleep restriction (SR: 5-h sleep opportunities); or weekend recovery (WR: simulated workweek of 5-h sleep opportunities followed by ad libitum weekend recovery sleep and then 2 days with 5-h sleep opportunities). Blood for metabolomics was collected on the simulated Monday immediately following the weekend. Nine machine learning models, including a machine learning ensemble, were built to classify samples from SR versus CON. Notably, SR showed decreased glycerophospholipids and sphingolipids versus CON. The machine learning ensemble showed the highest G-mean performance and classified 50% of the WR samples as insufficient sleep. Our findings show insufficient sleep and recovery sleep influence the plasma metabolome and suggest more than one weekend of recovery sleep may be necessary for the identified biomarkers to return to healthy adequate sleep levels.

## Introduction

In the United States, more than one-third of adults report sleeping less than the recommended seven hours per night and 30% report sleeping less than six hours per night^1–3^. Furthermore, since 2010, average reported sleep duration has decreased and the proportion of individuals reporting less than 7 h of sleep per night has increased^4, 5^. One common strategy to recover from insufficient sleep during work or school days is to catch-up or recover on sleep during weekends or free days^6, 7^. However, data informing the potential physiological responses to such weekend recovery sleep are mixed. Some data suggest weekend recovery sleep, compared with sleep restriction, can improve metabolic and inflammatory outcomes^8–10^. Alternatively, some data show weekend recovery sleep induces only partial recovery of the outcomes studied^11–14^, especially when repeating cycles of insufficient sleep and weekend recovery sleep are compared to adequate sleep. Identifying molecular biomarkers of insufficient sleep could help advance our understanding of the physiological responses to weekend recovery sleep and help improve sleep-based public health recommendations relevant to habitual sleep duration and recovery sleep.

Recent advances in machine learning and omics technologies, including metabolomics, have enhanced the biomarker discovery and development pipeline and have already been implemented in sleep research^15–17^. Unlike hypothesis driven strategies that target specific metabolites, and thereby potentially overlook unanticipated effects, untargeted metabolomics has the feature of assessing all metabolites detected in a sample.^18^ This often amounts to assessing thousands of metabolites simultaneously. Applying machine learning techniques to untargeted metabolomics data collected in studies with carefully controlled experimental sleep manipulation (restriction or extension) provides the opportunity to identify small molecules impacted by insufficient sleep. In a prior exploratory analysis of a within-participant cross-over study, we used this approach to identify a preliminary 65-compound biomarker signature of insufficient sleep^15^. Although promising, a critical next step in our line of research is to determine if similar approaches can be used to identify biomarkers of insufficient sleep in randomized, between-participant designs. Notably, biomarkers from between-participant designs will likely be more applicable to distinguishing between people with adequate versus insufficient sleep compared to within person changes that may not be as generalizable. Another important consideration to help advance the field is to understand if the performance of various machine learning algorithms differs when developing such biomarkers. Multiple machine learning algorithms have been combined to obtain superior modelling capacity in some prior studies, and these combined algorithms are named *machine learning ensembles*.^19, 20^ For example, various classifying models can be applied to the same dataset, then an algorithm compiles the outcomes of each model, and the “most voted” result is the outcome of the machine learning ensemble^21^. Identifying which machine learning algorithms have the best performance for developing metabolomics-based biomarkers of insufficient sleep is an essential step to inform the design of larger trials.

For the current study, we performed a secondary analysis using data collected in a prior three-group randomized controlled trial with control (CON), sleep restriction (SR), and sleep restriction with weekend recovery sleep (WR) groups^11, 22^. This study design facilitates between-group analyses to identify small circulating molecules that differentiate adequate versus insufficient sleep, thus building on our prior findings and helping inform larger more rigorous biomarker studies. Our main goals were to: (1) identify compounds that differentiate participants between 9h adequate versus 5h insufficient sleep opportunities in the laboratory, (2) define the performance of various machine learning models to predict 9h adequate versus 5h insufficient sleep using metabolomics data, and (3) to explore the impact of weekend recovery sleep by applying our top-performing biomarker to samples from WR participants who went through insufficient sleep followed by weekend recovery sleep.

## Materials and methods

### Participants

All study procedures were approved by the Colorado Clinical and Translational Sciences Institute, the Colorado Multiple Institutional Review Board (IRB), and the University of Colorado Boulder IRB, and were conducted in accordance to the Declaration of Helsinki. All participants signed the informed consent prior to initiating the study. Data were collected in a prior study from our group^11, 22, 23^. The current analyses included thirty-four healthy volunteers (17 women) with normal body mass index 22.4 ± 1.5 (mean ± SD) kg/m^2^, percent body fat 18.6 ± 6.3 in males and 32.1 ± 7.4 in females, and aged 25.3 ± 4.6 years. Detailed medical history, 12‐lead electrocardiogram, psychological and physical exam, sleep disorders screen, complete blood count, comprehensive metabolic panel, breath alcohol testing, and urine toxicology were performed to ensure the participants did not present any medical conditions and were drug free. All female participants were pre-menopausal, and we did not control the phase of menstrual cycle during study or limit contraceptive hormone use. Five women were on hormonal contraceptives (CON = 1, SR = 2, WR = 2). Eligibility criteria were detailed previously^11^.

### Protocol

**Figure 1:**
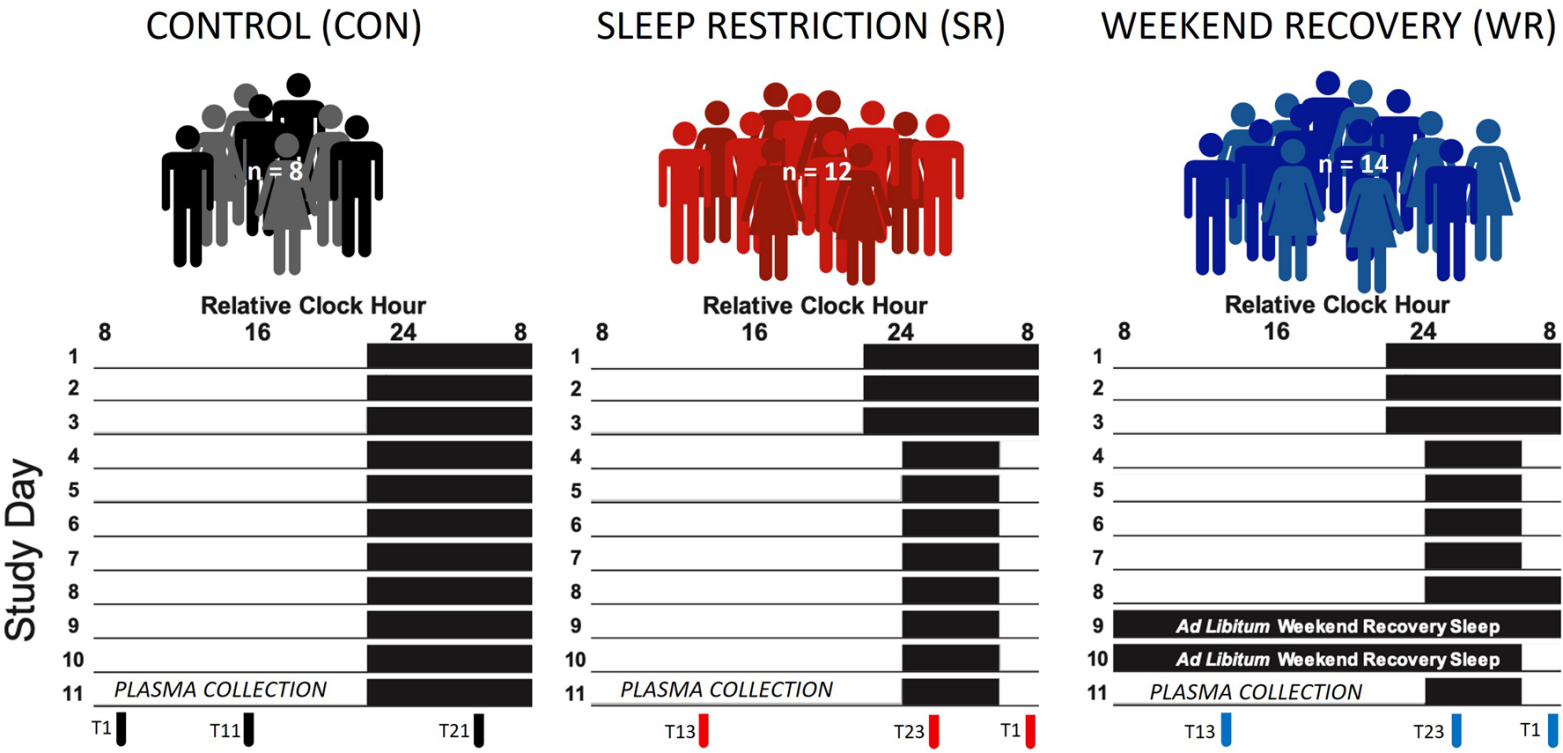
Study protocol. Scheduled wakefulness is represented by underlines and scheduled time in bed is represented by black boxes. In the WR group, sleep on study days 9 and 10 was ad libitum to simulate a weekend of recovery sleep. In the CON group, plasma samples were collected for metabolomics analyses on study day 11 at 1 h (T1), 11 h (T11), and 21 h (T21) after scheduled waketime. In the SR and WR groups, plasma samples were collected for metabolomics analyses on study day 11 at 1 h (T1), 13 h (T13), and 23 h (T23) after scheduled waketime. CON, control group; SR, sleep restriction group; WR, sleep restriction with weekend recovery group.

Participants were randomly assigned to either the CON (n = 8), SR (n = 12), or WR (n = 14) groups, balanced by sex. For 7 days prior to the in-laboratory experiment, participants were assigned individual sleep schedules with 9h sleep opportunities per night based on habitual sleep and waketimes. The in-laboratory experiment started with a 3-day baseline segment with 9h-sleep opportunities per night, aligned to each participant’s habitual sleep schedule. Each group underwent a different experimental protocol starting the night of study day 4 as follows. The CON group maintained 9h per night sleep opportunities. The SR group was provided 5h per night sleep opportunities. The WR group was provided sleep opportunities designed to simulate a workweek of insufficient sleep followed by a weekend with ad libitum sleep opportunities. Specifically, the WR group was scheduled to 5h per night sleep opportunities for study days 4–7. Study day 8 was the equivalent of a Friday with lights out scheduled 2h later than habitual lights out. Sleep opportunities on study days 8 and 9 had an enforced minimum of 10h time in bed. Days 9 and 10 were the equivalent of a Saturday and Sunday with self-selected waketimes and bedtimes with daytime napping permitted. WR participants were aware study day 11 would simulate a Monday with wake time scheduled 2h prior to habitual waketime. Blood samples for metabolomics analyses were collected on study day 11, 1 h (T1), 13 h (T13), and 23 h (T23) after scheduled waketime. For study days 11–13 the CON group maintained 9h sleep opportunities and the SR and WR groups maintained 5h sleep opportunities (Fig. 1). The complete study lasted until days 14–17 including recovery sleep prior to discharge from the laboratory in the SR and WR groups (not shown on Fig. 1). The participants were provided controlled diets designed to achieve energy balance during baseline and then were provided ad libitum food intake during the experimental segment of the study starting on day 4, as previously detailed^11, 22^.

*Polysomnography (PSG).* For the current analysis, PSG on study days 8–11 consisted of monopolar electroencephalography (EEG) electrodes referenced to contralateral mastoids (C3xA2, C4xA1, O1xA2, and F3xA2), chin electromyogram, electrocardiogram, and right and left electrooculograms as detailed previously^11^. PSG data were stored and sampled at 128 Hz with a 16 bit analog to digital converter. Scheduled wakefulness was monitored continuously, verified by laboratory staff and waking EEG.

*Slow wave activity (SWA).* For the WR group, SWA for power spectral analysis was calculated using the Fast Fourier Transformation on C3xA2 derivations with a MATLAB (Mathworks, Inc., vR2015a) program as detailed previously.^11^ Epochs scored as wakefulness or artifacts were not included in the power spectral analysis. Two-second Hanning windows averaged over each thirty-second epoch were used to estimate power at a 0.5 Hz resolution. High- and low-pass filters of 0.5 Hz and 25 Hz were applied to the data before analyses. In the delta range of 1–4 Hz across the entire sleep opportunity, cumulative SWA was measured for each individual and then averaged across individuals for the simulated weekend (study days 8–10, including naps for WR). For exploratory analyses, two variables were extracted for each participant in the WR group: (1) the sum and (2) the average per night of SWA for study days 8, 9 and 10.

*Insulin sensitivity.* Whole-body insulin sensitivity was quantified using a 3-stage hyperinsulinemic-euglycemic clamp on study day 12 as detailed previously.^11^

*Energy balance.* Average energy balance was calculated as energy intake minus energy expenditure across study days 11–13. All provided and uneaten food was weighed-back by registered dieticians to quantify energy intake. Twenty-four-hour whole-room indirect calorimetry was used to quantify energy expenditure as detailed previously^22^. After-dinner snack energy intake was quantified as all calories consumed after the provided dinner meal.

*Plasma Collection.* Blood was collected for metabolomics analyses on study day 11 at three time points: (1) fasting (T1; one hour after wake-time), (2) the approximate midpoint of scheduled wakefulness (T11 or T13), and (3) the approximate midpoint of each participant’s habitual sleep opportunity (T21 or T23). Thus, CON group blood draws were scheduled at 1 h, 11 h, and 21 h after scheduled waketime. SR and WR group blood draws were scheduled at 1 h, 13 h, and 23 h after scheduled waketime. Blood samples were collected into EDTA tubes and centrifuged (3600 RPM at 4 °C), the resulting plasma was aliquoted and stored at − 80 °C until the metabolomics analysis.

### Untargeted metabolomics

Methods for sample preparation, liquid chromatography, mass spectrometry, and data extraction were conducted similar to our previous work and described in Supplementary Material^15, 24, 25^. Metabolomics data were filtered to only include compounds detected in ≥ 50% of all samples. After filtering, the complete metabolomics dataset consisted of 22,236 features that corresponded to 3596 compounds. After filtering, missing values were imputed using the Bayesian Principal Components Analysis method in Metaboanalyst^26^, as in our prior work.^15, 27^ Metabolomics data were log2 transformed prior to statistical analyses.

### Compound annotation

First, for all compounds in the dataset we used Mass Profiler Professional (v.B.14.5; Agilent Technologies, Inc.) to putatively annotate common chemical names based on mass and isotope ratios with an error window of < 10 ppm. In this workflow, compounds are first searched against an in-house database of authentic standards followed by a search against an in-house database compiled from METLIN, human metabolome database (HMDB), Kyoto encyclopedia of genes and genomes (KEGG), and Lipid Maps. Second, we used MS/MS to generate additional annotation information for all compounds used in our biomarker models. The LC/MS chromatographic method was replicated for LC–MS/MS using 10, 20, and 40 eV collision energies on an Agilent 6545 Q-TOF, at a scan rate of 2 spectra/s, 1.3 m/z isolation width, and 30 s retention time window. Fragmentation data were matched to standards from the NIST 14 and 17 MS/MS spectral libraries.

### Compound selection for biomarker model development

**Figure 2:**
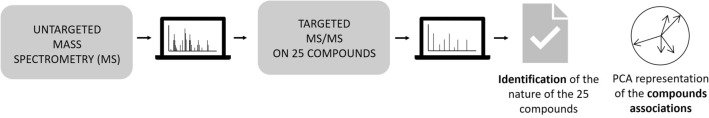
Process of identification of the top 25 compounds.

We detected a total of 3596 compounds. To identify the compounds that most effectively distinguish between the CON (9h per night sleep opportunities) versus SR (5h per night sleep opportunities) groups, we applied a random forest (RF) algorithm. We chose RF for its ability to handle and integrate such a high number of variables and rank them by importance scores^28^. RF works by randomly creating and testing classifying trees that integrate variables from the original dataset. When a tree correctly classifies the specified outcome, the variables composing that tree go up in the “variable importance ranking”. Thus, the most important variables in the ranking represent the metabolites that most strongly differentiate the CON and SR groups. Due to the random selection of variables (metabolites) in creating trees, metabolite rankings exhibit variability across iterations of applying the RF algorithm, particularly with smaller sample sizes. Thus, we repeated the RF algorithm 10,000 times, randomly assigning 50% of the participants to the training set and 50% to the test set. Variable importance scores for each compound were generated for each of these 10,000 iterations and then summed for an aggregate variable importance score. To determine the optimal number of compounds for building our final models, the RF was re-trained with 100 repeats using the top 5, 10, 15, 20, 25, 30, 35, 40, 45 and 50 compounds ranked by aggregate variable importance scores. The mean error rate was calculated for each selection of compounds. The minimum mean error rate reached an asymptote in the model with 25 compounds. Thus, adding more than 25 compounds to the model did not improve the mean error rate (Supplementary Fig. S1). As such, for subsequent biomarker model development we used these top 25 compounds as ranked by aggregate variable importance scores (Fig. 2).

### Biomarker model building

**Figure 3:**
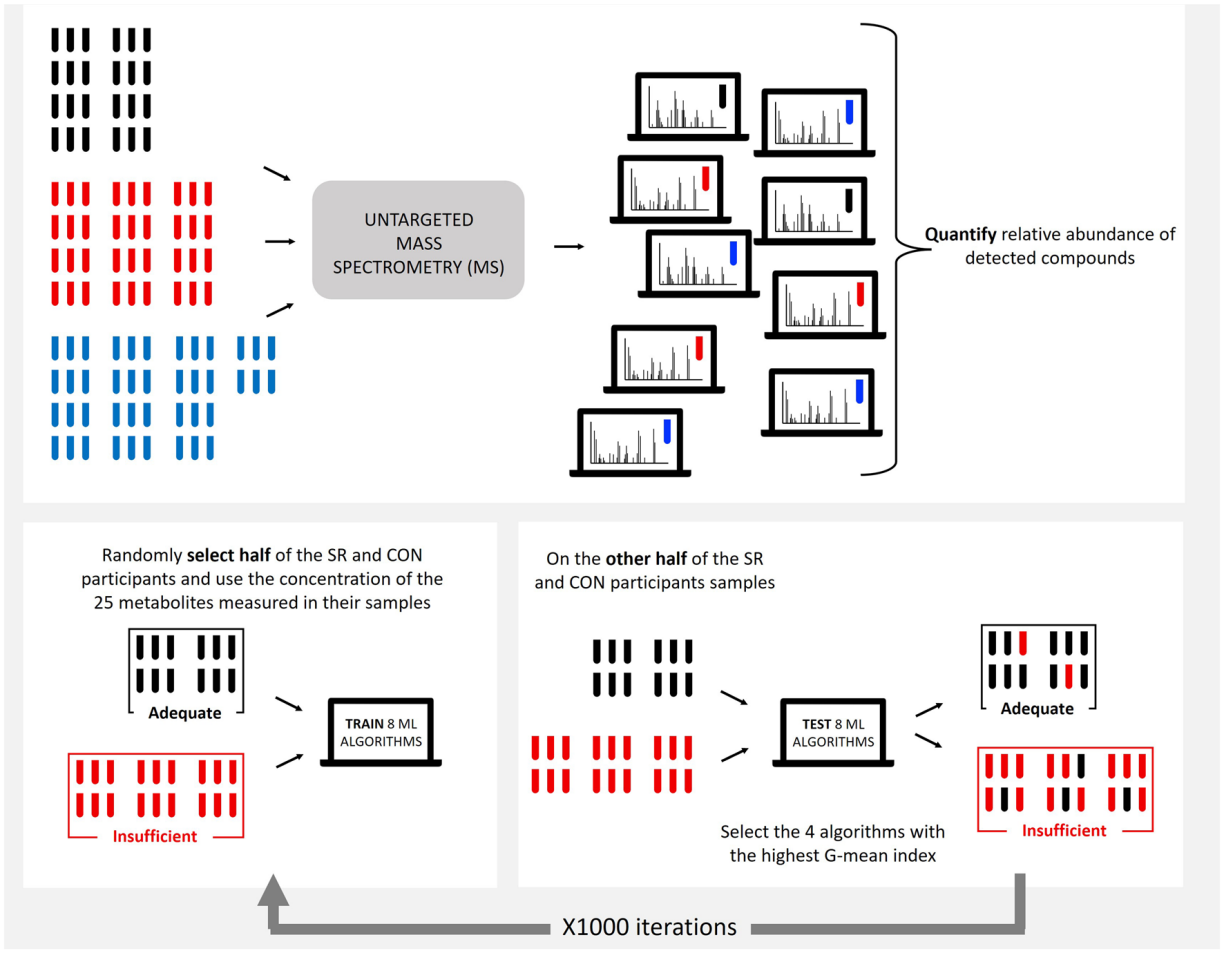
Schematic representation of the workflow used for biomarker algorithm development. Black sample indicators represent the CON group. Red sample indicators represent the SR group. Blue sample indicators represent the WR group.

Machine learning models perform differently based on the dataset and datatype they are applied to^29–31^. In our study, we aimed to assess the performance of eight machine learning algorithms. These algorithms were selected based on their relevance to metabolomics studies involving sleep and circadian outcomes, making them relevant and informative for our analysis. The eight algorithms were RF^32^, Support vector machine (SVM^15^), Key nearest neighbors (KNN^33^), Ordered logistic (polr^15^), Generalized Linear Model with Stepwise Feature Selection (glmnet^34^), linear discriminant analysis (lda^35^), partial least squares (pls^33^), and Recursive Partitioning and Regression Trees Accepts Case Weights (rpart^36^). We used the caret package (v6.0-90) in R to evaluate these eight machine learning algorithms to differentiate the CON versus SR groups using the identified top 25 compounds. For our approach, we randomly divided samples into training and testing sets for each algorithm in a loop repeated 1,000 times. In each loop, 50% of the CON and SR samples were used to train each algorithm and the other 50% used to test algorithm performance. The set of training and testing samples were randomly selected for each of the 1,000 iterations (Fig. 3).

### Performance metrics for biomarker models

Our objective was to build a balanced model capable of effectively distinguishing samples from participants in the SR group versus the CON group. Given the early stage of this research, we aimed for a model that equally prioritized sensitivity and specificity, which helps minimize bias from unbalanced sample sizes. A commonly used performance metric in this context is overall accuracy, which calculates the proportion of correctly classified samples. However, when study group sample sizes are unbalanced, this method can lead to bias in favor of the larger group. In our study, the SR group contained more samples (n = 34) than the CON group (n = 21). To prevent this issue, we used the G-Mean indicator, a frequently used performance metric in similar situations^37–39^. G-Mean is calculated as the square root of the product of sensitivity and specificity (G-Mean = √(sensitivity × specificity)). Unlike accuracy, G-Mean places equal importance on true negatives and true positives, minimizing potential bias from unbalanced group sample sizes. This choice of using the G-Mean metric ensures a balanced and robust assessment of the model's classification performance.

### Machine learning voting ensemble

Prior data shows combining machine learning models, called ensemble methods, can produce better classifiers than single models and their use in biomedical science is increasing^40^. As such, to generate an ensemble method we selected the four biomarker models with the highest mean G-mean indicators: KNN, RF, glmnet and SVM. In the ensemble, each individual biomarker model predicts sleep duration as adequate or insufficient and the most voted outcome is used.

### Biomarker model testing in the weekend recovery group

**Figure 4:**
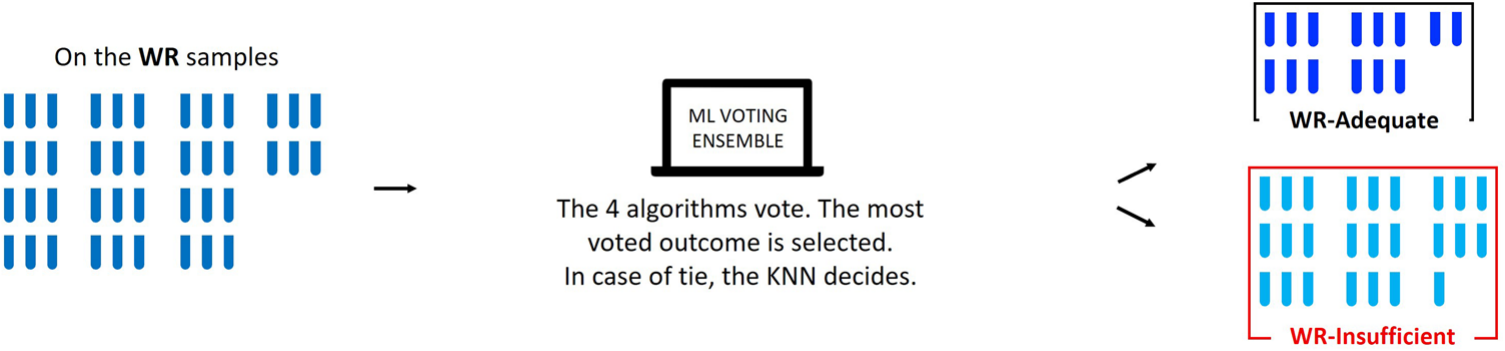
WR samples classification by a voting ensemble algorithm. WR, insufficient sleep with weekend recovery sleep group; ML, machine learning; WR-Adequate, samples from WR group scored as adequate sleep by ML ensemble; WR-Insufficient, samples from the WR group scored as insufficient sleep by ML ensemble.

Because the ensemble method produced the highest overall G-Mean, it was used to classify the WR samples (Fig. 4). We used principal component analysis (PCA) to graphically represent the samples by group: CON, SR, WR predicted as insufficient sleep (WR-INSUFFICIENT), and WR predicted as adequate sleep (WR-ADEQUATE). Wilcoxon tests were used to assess statistical significance of the differences in age, sex, total sleep time, SWA, insulin sensitivity, energy balance, and after-dinner snack energy intake between the WR samples classified as WR-ADEQUATE versus WR-INSUFFICIENT. Finally, because prior work from others and us identified diacylglycerol 36:3 as a potential biomarker of sleep restriction^15, 41^, we aimed to analyze the most closely related diacylglycerol in our current biomarker model. Although diacylglycerol 36:3 was not in our biomarker model, the closely related diacylglycerol 34:1 was. Thus, we analyzed diacylglycerol 34:1 using two-tailed t-tests to compare CON versus SR and CON versus WR.

## Results

The published primary findings from this study were focused on the effect of weekend recovery sleep on metabolic health outcomes including insulin sensitivity^11^ and energy balance^22^. Here, we conduct exploratory analyses of this randomized controlled trial on insufficient sleep and weekend recovery sleep by applying the techniques of machine learning and untargeted metabolomics on the plasma samples collected during the study.

### Top 25 compounds that differentiate adequate versus insufficient sleep

**Figure 5:**
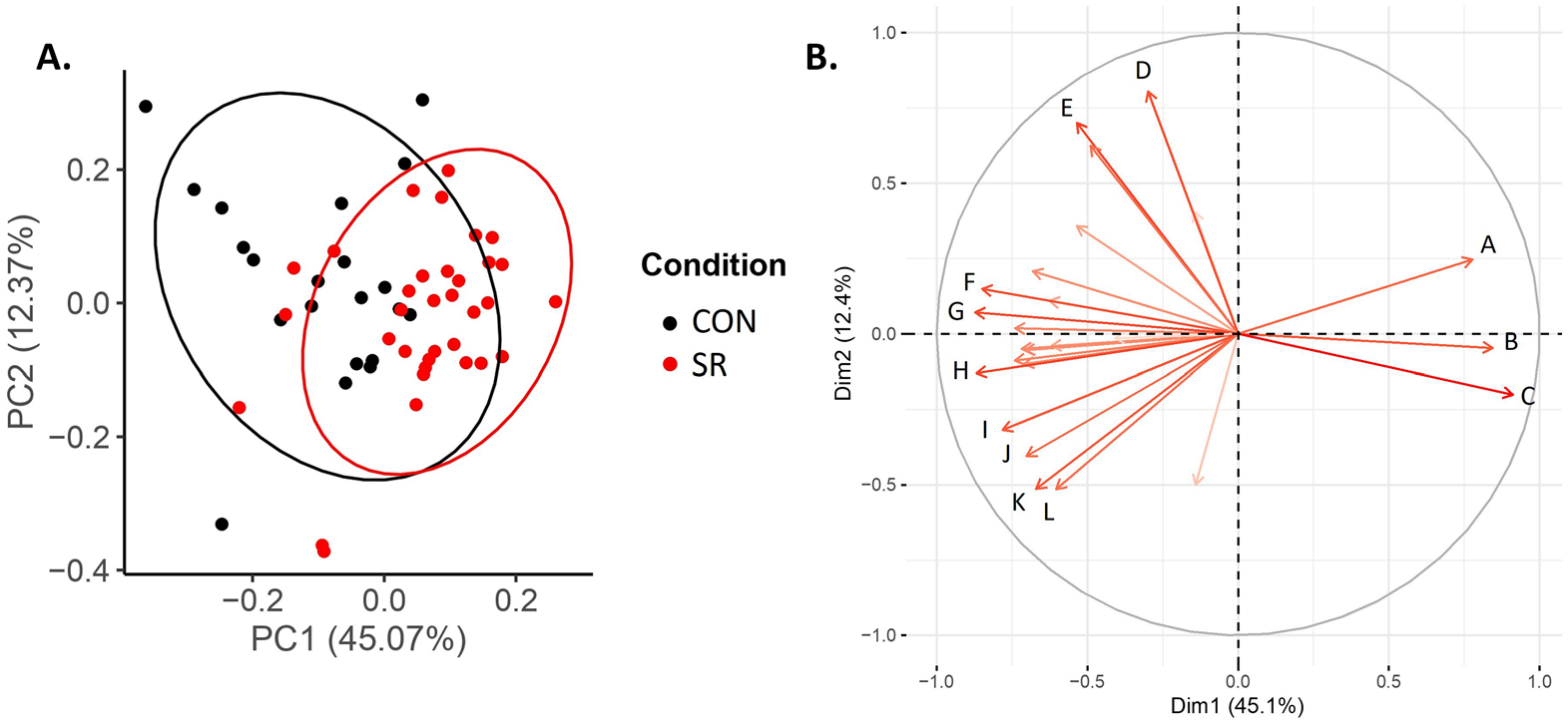
Principal Components Analysis of the SR and CON samples using the top 25 compounds. (**A**) PCA graph of the individuals. Ellipses level is 0.95 (**B**) PCA loadings plot of the 25 most important variables for SR and CON differentiation (A) Unknown; (B) Unknown; (C) Unknown; (D) PI(36:2); (E) Unknown; (F) PE(36:1); (G) *Unknown*; (H) PE(38:1); (I) PC(P-38:3); (J) PA(P-40:0); (K) PE(O-33:0); (L) PE-Cer(d38:2).

Out of the top 25 compounds identified as most important to differentiate samples from the CON group versus SR group (Fig. 2, Supplementary Table S1), 1 compound is from the aqueous phase and 24 compounds are from the lipid phase. The aqueous compound was not identified through MS/MS. Among the 24 lipid compounds, 14 had positive matches to MS/MS spectral libraries. Broadly, the lipid compounds included diacylglycerols, glycerophospholipids, and sphingolipids. The PCA in Fig. 5A graphically indicates the clustering of CON and SR samples using these top 25 compounds. Combined, Principal Component 1 and Principal Component 2 reflect ~ 57% of the variability in the data, with the CON and SR groups showing visual separation primarily along Principal Component 1.

The loadings plot (Fig. 5B) derived from the PCA analysis helps visualize which compounds influence each Principal Component and thus help characterize the difference between the CON and SR groups. Accordingly, the SR group showed higher abundance for the unidentified compounds, whereas the CON group presented a higher abundance in the compounds identified as glycerophospholipids, glycerolipids, and sphingolipids. Additionally, one of the 25 compounds included the diacylglycerol 34:1, which was lower (*p* < 0.05) in the SR compared to CON group. Prior work^41^, including from us^15^, identified decreased diacylglycerol 36:3 as a potential biomarker of sleep restriction, we therefore present the similar changes we observed for diacylglycerol 34:1 in Supplemental Fig. S3.

### Biomarker model performance

**Figure 6:**
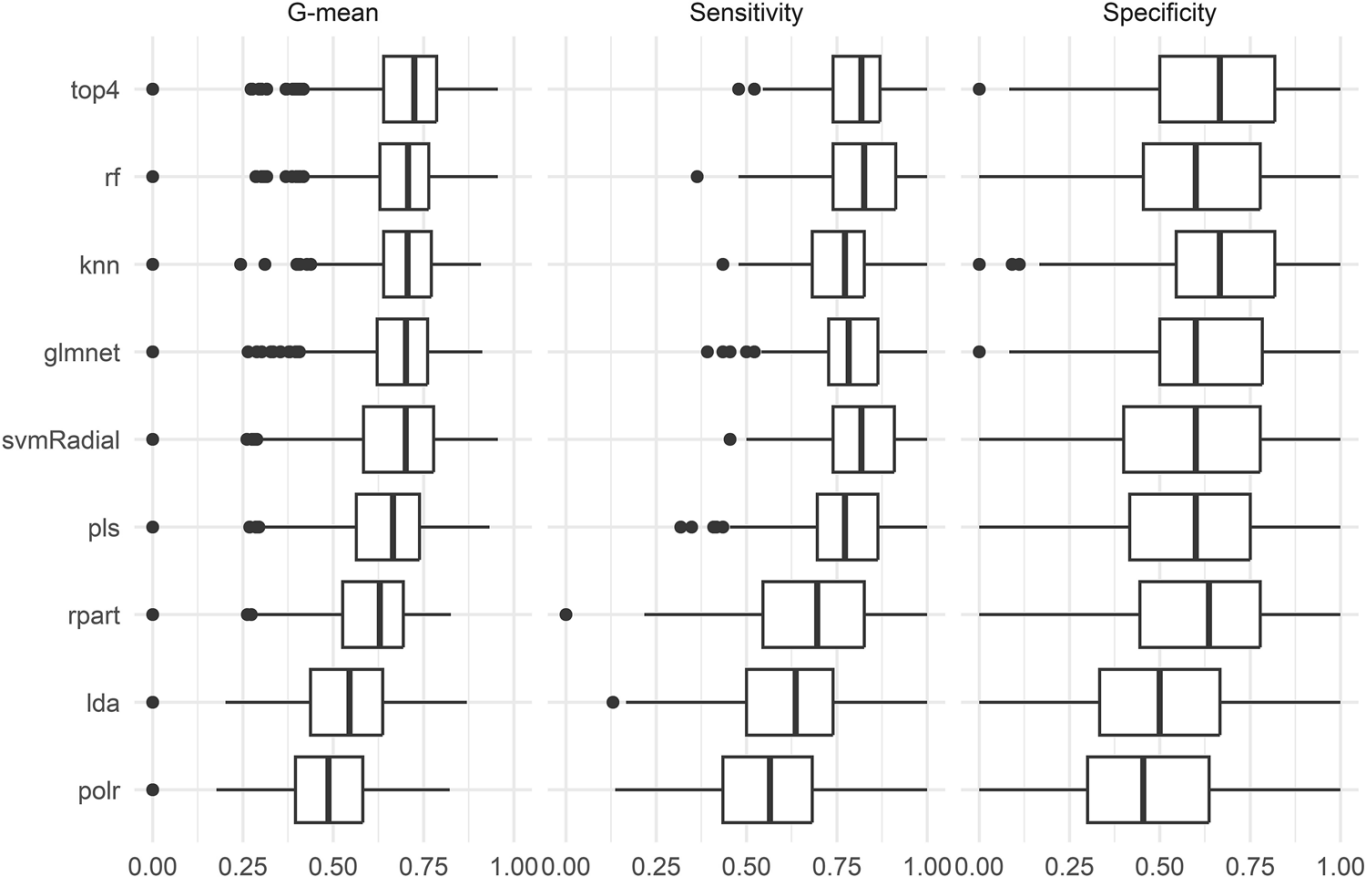
G-mean, sensitivity and specificity for each machine learning algorithm trained and tested on sample from the CON and SR groups.

G-mean values, sensitivity, and specificity from the 8 machine learning algorithms are represented in Fig. 6 and Supplementary Table S2.

The four algorithms with the highest G-means (glmnet, knn, rf, svm) were selected to build an ensemble. The ensemble model produced the highest overall G-mean out of all models. Regarding the classification, out of the CON group samples, 15 out of 21 (71%) were correctly classified. Of the SR group samples, 29 out of 34 (85%) were correctly classified (see Supplementary Fig. S2 for individual participant classifications).

### Classification of samples from the WR group

When the ensemble algorithm was applied to samples from the WR group, 22 (55%) samples were classified as adequate sleep (WR-ADEQUATE) and 18 (45%) samples were classified as insufficient sleep (WR-INSUFFICIENT).

**Figure 7:**
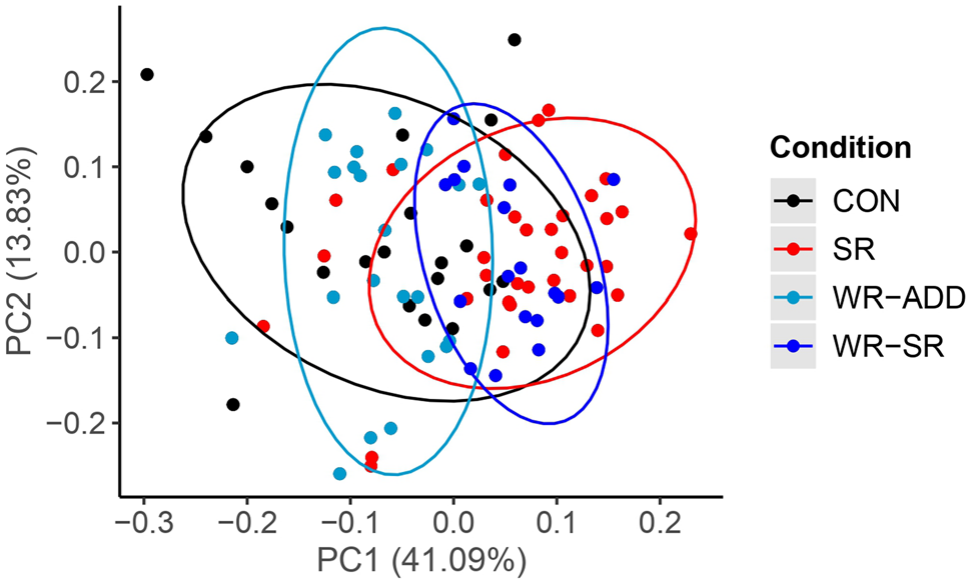
Principal component analysis of the CON, SR, and WR samples. The color of samples from the WR group denotes how the ensemble algorithm classified each sample with light blue as WR-ADEQUATE (WR-ADD) and dark blue as WR-INSUFFICIENT (WR-SR). Ellipses level is 0.95.

The PCA representation (Fig. 7) shows the samples from the WR group are generally located at the intersection of samples from the CON and SR groups. When colored according to the classification made by the ensemble, the WR samples classified as WR-ADEQUATE overlap with the CON samples whereas the WR samples classified as WR-INSUFFICIENT overlap with the SR samples.

**Figure 8:**
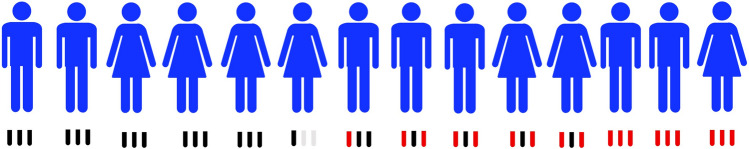
Schematic representation of the classification of the samples of the participants from the WR group. The color of tik marks denotes how the ensemble algorithm classified each sample with black as WR-ADEQUATE, red as WR-INSUFFICIENT, and grey for the missing samples, the sex of the participant is symbolized by the shape of the character. For each participant, the first sample was collected at 1 h, the second at 13 h, and the third at 23 h after scheduled wake time.

**Figure 9:**
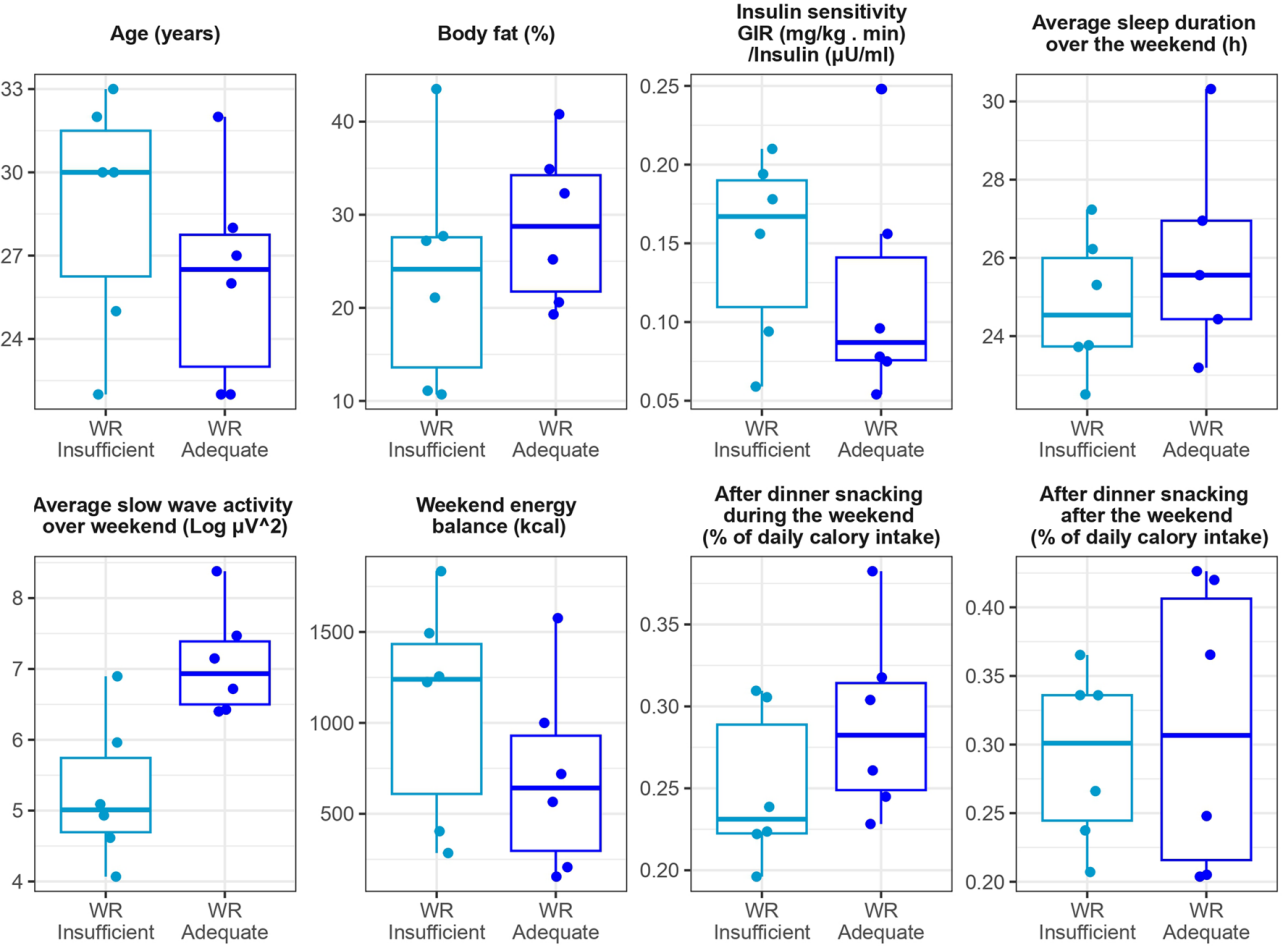
Exploratory comparison of characteristics from individuals with samples either classified as “insufficient” or “adequate” sleep after a weekend recovery. A participant was included in the WR-insufficient group if at least two of their three samples were classified as insufficient sleep, and the same logic was used to compose the WR-adequate group.

The classification of samples for individual WR participants are shown in Fig. 8. Out of the 13 participants with complete data, all samples from 5 participants were exclusively classified as WR-ADEQUATE, all samples from 3 participants were exclusively classified as WR-INSUFFICIENT, and 5 participants had mixed classifications with at least one sample classified as WR-ADEQUATE and one sample classified as WR-INSUFFICIENT. These results suggest potential interindividual variabilities in the effect of weekend sleep recovery on the metabolome. Among the 5 WR participants with mixed classifications of samples, the first sample collected at T1 was always classified as WR-INSUFFICIENT. In contrast, the samples collected at T13 were classified as WR-ADEQUATE in 3 out of 5 cases, and the samples collected at T23 were classified as WR-INSUFFICIENT in 4 out of 5 cases. Notably, the timing interval between food intake and sample collection was shortest for the T13 samples, which were mostly classified differently than the T1 and T23 samples. This suggests these mixed classifications between timepoints within participants may be influenced by food intake, with T1 and T23 producing more consistent classifications. Unfortunately, the WR study group is too small to conduct meaningful in-depth analyses of the origins of these potential differences. However, as additional exploratory analyses to help inform potential future investigations, we plotted the following characteristics of WR participants, classified as WR-INSUFFICIENT versus WR-ADEQUATE: age, body fat percentage, insulin sensitivity, sleep duration over the weekend preceding the blood collection, average SWA over the weekend, energy balance, and after dinner snacking (Fig. 9). These pilot analyses suggest that more SWA ﻿(*p* < 0.05) during a weekend of recovery sleep could be associated with better recovery of our biomarker model.

## Discussion

We used data from a rigorous randomized controlled trial to determine if a blood metabolomics-based biomarker can distinguish between participants that received a week of adequate sleep versus a week of insufficient sleep. We also examined the impact of a weekend of recovery sleep on our top-performing biomarker. Our final biomarker model consists of 25 plasma compounds and is designed to differentiate participants between 7 nights of 9h versus 5h sleep opportunities. As part of our biomarker development and analysis workflow we compared the performance of 8 different machine learning algorithms. The top four algorithms, based on G-mean, were Random Forest, Key Nearest Neighbors, Support Vector Machine and Generalized Linear Model with Stepwise Feature Selection. These four algorithms had similarly better G-mean values compared to the bottom four performing algorithms, highlighting the importance of considering different machine learning algorithms in future study design. Using these four best-performing algorithms, we built a voting ensemble which performed better than the individual models based on G-mean. This final ensemble model showed a similar range of accuracy as our previously published metabolomics-based biomarker of insufficient sleep^15^. Importantly, our prior biomarker was developed from data collected in a cross-over study whereas our current biomarker was developed from a randomized controlled trial with independent CON and SR study groups. These findings provide proof-of-principle evidence that it is possible to use small molecules in the plasma to differentiate individuals between adequate versus insufficient sleep opportunities in the laboratory.

Twenty-four of the 25 compounds in our biomarker model are lipids, primarily consisting of glycerophospholipids and sphingolipids. Comparing the compounds in the current biomarker panel with the compounds in our previously published biomarker panel, the broad compound class representations of glycerophospholipids and sphingolipids were similar, and two compounds are common between the two studies: SM(43:2) and PE(35:3), each showing decreased abundance during sleep restriction versus adequate sleep. Unfortunately, due to a large number of unidentified compounds in our prior 65-compound biomarker signature of insufficient sleep it is not possible to directly evaluate the performance of that biomarker signature in this dataset. This issue exists across untargeted metabolomics studies and highlights the need for development of targeted biomarker assays in large-scale clinical trials. Despite limitations, we compare our biomarker compounds against prior metabolomics-based biomarkers of sleep duration. Several prior metabolomics studies with total sleep deprivation have been published. In a study with twelve healthy participants that were given controlled meals, the metabolome was analyzed every two hours during 24 h of total sleep deprivation. Compared to the first day with an 8h sleep opportunity, several plasma glycerophospholipids and sphingolipids were significantly increased during total sleep deprivation, opposite the direction of our findings^42^. In another study, the impact of 40 h of total sleep deprivation on lipid metabolism was assessed in twenty male participants undergoing a constant routine with hourly equicaloric snacks. Out of the 263 lipids analyzed, four reliably decreased and 21 reliably increased in at least half of the participants during total sleep deprivation^43^. Another study in twelve healthy young female participants with controlled meals also analyzed the impact of total sleep deprivation on plasma metabolites^44^. In that study, 15 out of the 130 metabolites analyzed, including glycerophospholipids, varied significantly during total sleep deprivation versus baseline.^44^ Based on these prior studies and our present data, plasma lipids are broadly altered during sleep restriction with some increasing and others decreasing. Directionally, these changes are not always consistent between total sleep deprivation lasting up to 40 h versus partial sleep restriction lasting 5–8 days, suggesting these different models of experimental sleep manipulation likely have different physiological consequences.

Metabolomics analyses from studies of experimental sleep restriction have also been published. In a unique study with parallel sleep restriction in rats and humans (n = 10; 5 women), 5 days of experimental sleep restriction was compared against baseline adequate sleep. A global shift in lipid compounds was observed, with a noted decrease in oxalic acid and diacylglycerol 36:3 in both rats and humans following sleep deprivation with ad libitum access to food^41^. Of note, we previously reported a similar decrease in diacylglycerol 36:3 following 5 nights of experimentally imposed insufficient sleep in humans^15^. Although neither oxalic acid or diacylglycerol 36:3 were in our current biomarker model, the similar diacylglycerol 34:1 was in our biomarker model and showed a reduction in SR versus CON (Supplementary Fig. S3), consistent with previous observations for diacylglycerol 36:3. These data provide further evidence that experimental sleep restriction may result in altered diacylglycerol metabolism. Another study was performed on eleven healthy adults that completed two sessions of eight nights each, one with a 5.5 h sleep opportunity and one with an 8.5 h sleep opportunity, with controlled meals. The samples resulting from the sleep restriction session presented globally higher levels of several fatty acid, bile acid, steroid hormone, and tricarboxylic acid cycle intermediates and lower levels of glucose, some monosaccharides, gluconate, and five-carbon sugar alcohols^45^. Finally, in a cross-sectional analysis of metabolomics data from the Nurses’ Health Study and Women’s Health Initiative cohorts, self-reported short sleep duration was associated with higher levels of several lysophospholipids and lower levels of diacylglycerol 34:3 and 11 triacylglycerols^46^.

In general, findings of these prior studies and the present work have in common the identification of an impact of total sleep deprivation and sleep restriction on plasma metabolomics, and most commonly an effect on lipids. However, the specific findings are mixed, sometimes appearing contradictory, which may be explained by the diversity in targeted versus untargeted metabolomics methods, timing of sample collection, controlled versus ad libitum food availability, medications and drugs, experimental sleep restriction protocols, sleep laboratory settings, methods of statistical analysis, and participant characteristics including age, sex, and health status. This also emphasizes that the precise pathways through which sleep restriction may impact lipid metabolism remain to be identified and likely include many different classes of lipids.

We further assessed our biomarker model by examining the impact of a workweek of insufficient sleep followed by weekend recovery sleep, using completely naive samples from the WR group that did not contribute to model training. Overall, ~ 55% of the samples from the WR group were classified as adequate sleep and ~ 45% classified as insufficient sleep. These data are visually represented by the PCA (Fig. 5) where there was clear overlap between the WR-ADEQUATE and CON samples and then the WR-INSUFFICIENT and SR samples. These findings indicate that a single weekend of recovery sleep is unlikely to fully compensate the adverse health consequences from a workweek of insufficient sleep, at least from a metabolomics perspective. This underlines the existence of potential interindividual differences in response to recovery sleep. We explored some potential leads that could help explain these differences in response to weekend recovery sleep: age, body fat percentage, insulin sensitivity, sleep duration over the weekend preceding the blood collection, average SWA over the weekend in response to sleep restriction during the workweek, energy balance, and after dinner snacking. Regarding body fat percentage, there are established differences between men and women. A bigger cohort is needed to fully study potential sex differences and links between body composition and weekend recovery sleep. From these exploratory assessments, the SWA during weekend recovery sleep following a workweek of insufficient sleep appears as a relevant lead to follow-up on in future work. Notably, given the variability in SWA data in general, studies with a higher number of participants will be necessary to test this hypothesis. Another parameter requiring further investigation, particularly with a larger sample size, is the potential influence of time-of-day and fasting duration on our biomarker model. In our study, five WR participants had samples classified as both WR-ADEQUATE and WR-INSUFFICIENT. As previously noted, the T13 sample collected in the middle of the day showed the most heterogeneity in classification compared to the T1 and T23 samples. This variability may be due to the relatively short fasting duration prior to collecting T13, or it could be influenced by the circadian timing of sample collection. We previously developed a metabolomics-based biomarker of circadian phase, and none of the identified metabolites in our current analysis overlapped with those from our circadian biomarker^27^. However, there is overlap in metabolite classes including several glycerophospholipids and diacylglycerols. To thoroughly investigate the potential influence of circadian timing on our biomarker model, future studies using established circadian protocols, like the constant routine, are needed.

Additionally, it is worth noting that in clinical practice, blood collections typically occur in the fasted state. As the field progresses, it is conceivable that most blood draws will occur in the morning fasted state in clinical settings. Consequently, any variation in performance of samples collected across the day may have less clinical relevance. Intriguingly, if we focus exclusively on the fasting T1 samples for the WR participants, the overall classification for WR-ADEQUATE and WR_INSUFFICIENT would change for just one participant. Consequently, our exploratory analyses in Fig. 9 would remain nearly unchanged, with SWA during the weekend still emerging as a potentially important factor that could contribute to the recovery of our biomarker model.

There are important limitations to the current studies on sleep and the metabolome, including our present study. First, many findings are based on a combination of fasting and non-fasting samples and it is not precisely defined how fasting status may impact the biomarkers. Ideally, large clinical trials will consistently use fasting samples to minimize this potential source of variability. Second, we previously showed a delay in circadian melatonin timing in the SR and WR groups following the sleep restriction and weekend recovery portions of the protocol but no change in the CON group^11^. This differential shift in circadian timing between groups means samples were collected at difference circadian phases and this may have contributed to differences in our biomarkers between groups. Third, larger sample sizes are needed to produce more rigorous statistical analyses that include completely independent biomarker validation and testing cohorts. Fourth, many of the published studies are based on different metabolomics platforms and methodologies including targeted and untargeted approaches. Such differences make it challenging and impossible in some cases to directly compare results between studies. As the field progresses, use of targeted assays with absolute quantification and compound annotation based on established standards will help enhance the ability to identify compounds with similar responses across different studies and cohorts. Fifth, most of the currently published data are derived from laboratory studies with acute (days to weeks) experimental sleep restriction in healthy young and lean adults. It is unclear how or if data from such experimental sleep restriction studies translates to people with real-world habitual short sleep duration outside the laboratory. Sixth, most of the published biomarkers, including our current model, lack independent performance evaluation in completely naïve testing data sets. Optimally, such performance evaluations will be conducted in multiple labs across multiple cohorts. Because rigorous biomarker performance validation/evaluation is a large and time-consuming endeavor, a more acute next step is to begin translating the current findings into free-living people with habitual adequate and insufficient sleep. Such trials will have more noise in the signal and require much larger sample sizes but will have the advantage of greater generalizability to a broader population. Time and economic investments in performance evaluation studies are likely better invested in these more generalizable studies over the long-term. We expect that studies with larger sample sizes that consist of individuals with objectively defined habitual short or adequate sleep duration, over months to years, would help develop more robust biomarker models. Furthermore, such investigations could help improve our understanding of the effect of habitual short sleep duration on adverse cardiometabolic health outcomes. In parallel, such knowledge could also inform new countermeasure strategies to combat the adverse health consequences associated with habitual short sleep duration.

In conclusion, our findings provide additional evidence that the human plasma metabolome is affected by insufficient sleep, including variations of lipid levels such as glycerophospholipids, sphingolipids, and diacylglycerols. These compounds can be used to develop machine learning models that differentiate samples between adequate and insufficient sleep using a between-participants laboratory-controlled design. These findings help set the stage for larger fully powered trials to identify biomarkers that differentiate people between habitual adequate sleep versus habitual insufficient sleep. Finally, our data further suggest weekend recovery sleep may only partially compensate the effects of a workweek of insufficient sleep and highlight potential interindividual differences in the physiological response to weekend recovery sleep.

### Supplementary Information

Supplementary Information.

## Data Availability

The datasets generated during and/or analyzed during the current study are available from the corresponding author on reasonable request.
