# Supplementary material for: Insufficient sleep and weekend recovery sleep: classification by a metabolomics-based machine learning ensemble
Source: Sci Rep. 2023 Nov 30;13:21123. doi: 10.1038/s41598-023-48208-z (PMC10689438; doi:10.1038/s41598-023-48208-z)
Supplement: Supplementary file 1 — Supplementary Information. [file 41598_2023_48208_MOESM1_ESM.pdf]

SUPPLEMENTARY MATERIALS

Title: *Insufficient sleep and weekend recovery sleep: classification by a metabolomics-based machine learning ensemble***.**

Authors and Author Affiliations:
Marie Gombert[1,2], Nichole Reisdorph[3], Sarah J. Morton[4], Kenneth P. Wright Jr.[4,5]*, and Christopher M. Depner[4,6]*
[1] Department of Pediatrics, Obstetrics and Gynecology, University of Valencia, 46010 Valencia, Spain.
[2]Center for Health Sciences, SRI International, Menlo Park, CA, USA
[3]Skaggs School of Pharmacy and Pharmaceutical Sciences, University of Colorado Anschutz Medical Campus, Aurora, CO, USA
[4]Sleep and Chronobiology Laboratory, Department of Integrative Physiology, University of Colorado Boulder, Boulder, CO, 80309, USA
[5]Division of Endocrinology, Metabolism, and Diabetes, University of Colorado Anschutz Medical Campus, Aurora, CO, 80045, USA
[6]Department of Health and Kinesiology, University of Utah, Salt Lake City, UT, 84112, USA
*Corresponding authors

Untargeted Metabolomics Sample Preparation

All solvents used for metabolomics workflows were of liquid chromatography/mass spectrometry (LC/MS)-grade as follows: water and isopropyl alcohol from Honeywell Burdick & Jackson (Muskegon, MI, USA); methyl tert-butyl ether from J.T. Baker (Central City, PA, USA); acetonitrile, methanol and formic acid from Fisher Scientific (Fair Lawn, NJ, USA); standards from Avanti Polar Lipids Inc. (Alabaster, AL, USA) and Sigma Aldrich (St. Louis, MO, USA); glass pipette tips, plastic pipette tips and microcentrifuge tubes from Fisher Scientific (Fair Lawn, NJ, USA); Pyrex glass culture tubes from Corning Incorporated (Corning, NY, USA). To create quality control (QC) samples, aliquots from multiple participants were pooled and re-aliquoted. Pooled samples served as sample preparation and instrument QCs.

For sample preparation, 100 μL per sample was transferred to a 1.5 mL microcentrifuge tube at 0°C. 10 μL of hydrophobic and hydrophilic standards and spikes

were added to each sample. To precipitate proteins, 400 μL of ice-cold methanol was added to each tube and then centrifuged for 15 min at 0°C at 18,000 x g. The resulting supernatant was then dried in glass culture tubes under N2 at 35°C for ~1h. After drying, 3 mL of methyl tert-butyl ether (MTBE) and 750 μL of water was added to each glass culture tube and then tubes were centrifuged for 10 min at room temperature at ~200 x g. The resulting MTBE layer (hydrophobic fraction) was transferred to a clean glass culture tube and the remaining layer was the hydrophilic fraction. This process was again repeated with 3.0 mL of MTBE added to the remaining hydrophilic fraction. The resulting MTBE layer was aspirated and combined with the first MTBE layer. These combined MTBE fractions were dried under N2 at 35°C and re-suspended in 200 μL methanol. Each sample (hydrophobic fraction) was finally transferred to a glass auto-sampler vial and stored at -80°C until analysis.

For the remaining hydrophilic fractions, samples were dried under N2 at 35°C and then 100 μL of water and 400 μL of ice-cold methanol were added and then centrifuged at ~200 x g for 1 min. Supernatants from each sample were transferred to a 1.5 mL microcentrifuge tube and then stored at -80°C for 25 min then centrifuged for 15 min at 0°C and 18,000 x g. The resulting supernatant was transferred to a new 1.5 mL microcentrifuge tube and dried in a vacuum centrifugal concentrator at 45°C and re-suspended in 100 μL of 95:5 water: acetonitrile. Finally, each sample was transferred to a glass auto-sampler vial and stored at -80°C until analysis.

Liquid Chromatography

The hydrophobic fraction was separated prior to mass spectrometry using an Agilent

Zorbax Rapid Resolution HD SB-C18, 1.8 micron, 2.1 x 100 mm analytical column on an Agilent 1290 series pump using a 4µL injection volume. HPLC flow rate was 0.7 mL/min. Mobile phase A was water with 0.1% formic acid. Mobile phase B was 60:36:4 isopropyl alcohol:acetonitrile:water with 0.1% formic acid. The gradient for positive mode was: 0.0–1.0 min 30–70% B, 1.0–7.92 min 70–100% B, 7.92–10.4 min 100% B, 10.4–10.5 min 100–30% B, 10.5–15.1 min 30% B. Autosampler tray temperature was 4°C and column temperature was 60°C. The hydrophilic fraction was separated prior to mass spectrometry using an Agilent 1200 series pump using a Phenomenex Kinetex HILIC, 2.6 µm, 100 Å (2.1 × 50 mm) analytical column and an Agilent Zorbax Eclipse Plus-C8 5 µm (2.1 × 12.5 mm) narrow bore guard column. Autosampler tray temperature was 4°C, column temperature was 20°C, and injection volume was 1 µL. The flow rate was 0.6 mL/min. Mobile phase A was 50% ACN with pH 5.8 ammonium acetate, and mobile phase B was 90% ACN with pH 5.8 ammonium acetate. Gradient elution: 0.2 minutes 100% B, 0.2–2.1 minutes 100–90% B, 2.1–8.6 minutes 90–50% B, 8.6–8.7 minutes 50–0% B, 8.7–14.7 minutes 0% B, 14.7–14.8 minutes 0–100% B, 14.8-24.8 minutes 100% B.

Mass Spectrometry (MS)

Hydrophobic fraction conditions were: Agilent 6220 Time-of-Flight (TOF)- MS with dual ESI source, scan rate 2.02 spectra/s, mass range 60–1600 m/z, gas temperature 300°C, gas flow 12.0 L/min, nebulizer 30 psi, skimmer 60V, capillary voltage 4000V, fragmentor 120V, reference masses 121.050873 and 922.009798 (Agilent reference mix). Hydrophilic fraction conditions were: Agilent 6520 Quadrupole Time-of-Flight mass spectrometer (Q-TOF-MS) in positive ionization mode with ESI source, mass range 50–1700 m/z, scan rate 2.21, gas temperature 300°C, gas flow 10.0L/min, nebulizer 30 psi, skimmer 60V, capillary

voltage 4000V, fragmentor 120V, reference masses 121.050873 and 922.009798 (Agilent reference mix).

Supplementary Figures

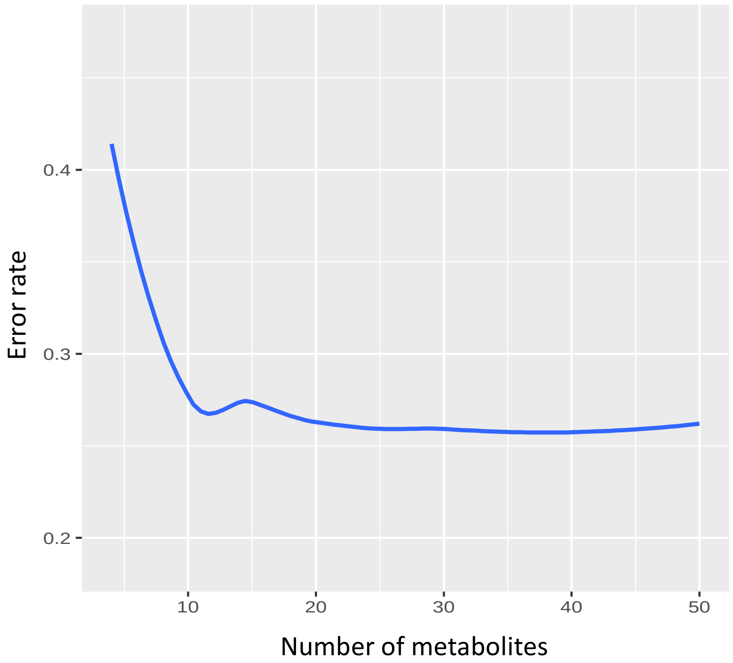

Supplementary Figure 1. Error rate of the Random Forest algorithm according to the number of compounds included. The curve binds the means of the simulations using a locally weighted polynomial (LOESS).

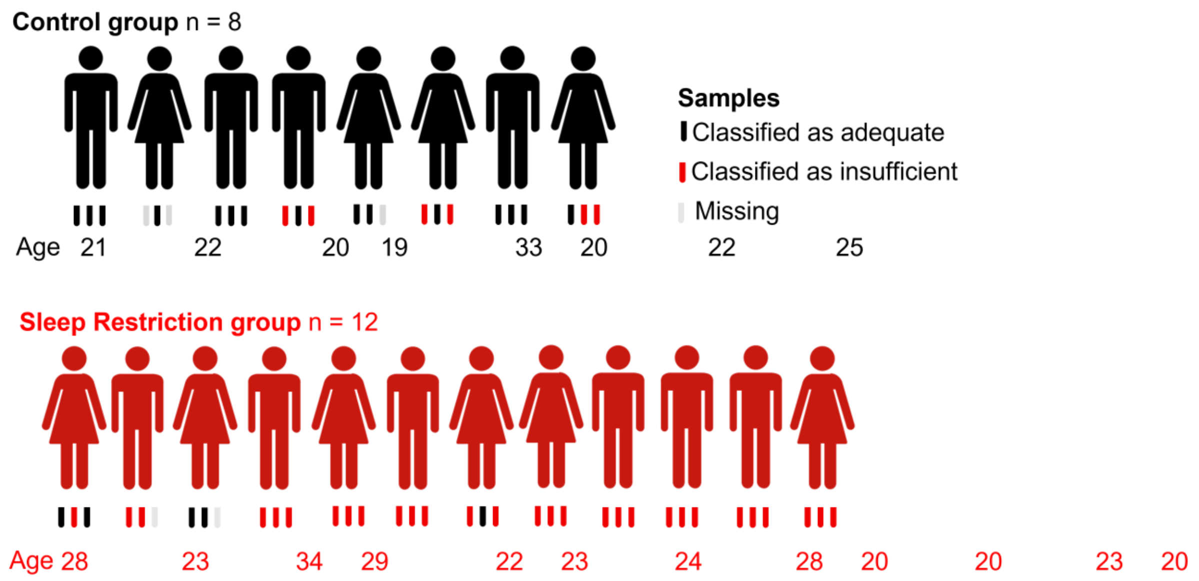

Supplementary Figure 2. Representation of the classification of the samples by study participant

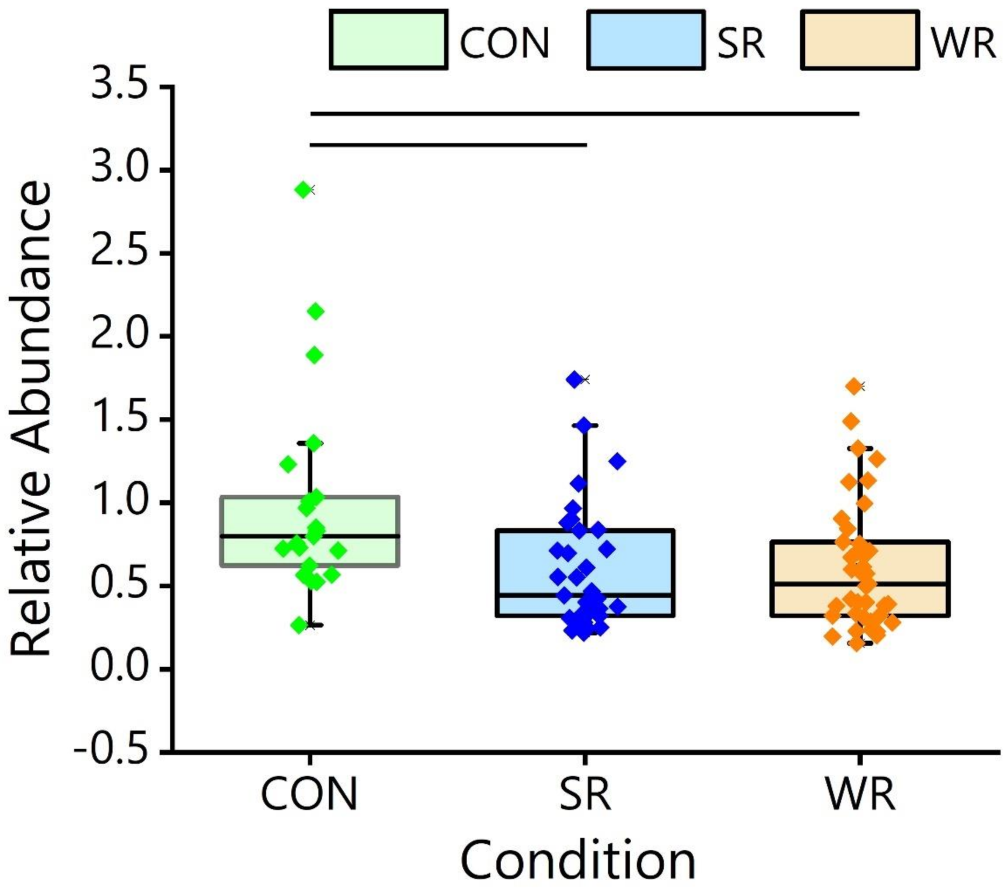

Supplementary Figure 3. Relative abundance of diacylglycerol 34:1. Solid horizontal black lines at top of figure represent significant differences ($p<0.05$) between study groups at ends of the line. The upper and lower bounds of boxes demarcate the 25%-75% of data, the horizontal line through boxes represent the median, and error bars represent 1.5*interquartile range. CON, control group; SR, sleep restriction group; WR, weekend recovery group.

Supplementary Table 1. Descriptive table of the 25 MS/MS compounds identified as most different between SR and CON groups and used to build the model.

Compound	Mass	Retention time	Accession ID	Category	ID	Importance ranking	CON	SR	WR
LysoPE(18:0)	481.3173	1.8129948	PubChem CID 9547068	Glycerophospholipids [GP]	MSI 2	4	15.8 (15.32 - 15.99)	15.42 (15.13 - 15.69)	15.59 (15.3 - 15.69)
PA(P-40:0)	744.6024	6.5319886	PubChem CID 52929707	Glycerophospholipids [GP]	MSI 2	6	15.77 (15.64 - 15.93)	15.26 (15.02 - 15.54)	15.68 (15.35 - 15.89)
PC(45:1)	767.5494	5.578987	PubChem CID 52923219	Glycerophospholipids [GP]	MSI 2	2	16.23 (15.92 - 16.57)	15.86 (15.71 - 16)	16.02 (15.81 - 16.36)
PC(45:4)	767.5472	5.593995	PubChem CID 52923219	Glycerophospholipids [GP]	MSI 2	3	16.19 (15.89 - 16.58)	15.78 (15.52 - 15.99)	16.04 (15.81 - 16.36)
PE-Cer(d38:2)	714.5659	5.235992	PubChem CID 70699010	Sphingolipids [SP]	MSI 2	25	14.7 (14.62 - 14.83)	14.37 (14.18 - 14.62)	14.51 (14.31 - 14.78)
PE(36:4)	767.5481	5.910008	PubChem CID 52924235	Glycerophospholipids [GP]	MSI 2	5	16.44 (16.39 - 16.56)	16.22 (16 - 16.3)	16.32 (16.21 - 16.43)
PE(36:1)	745.5567	6.239012	PubChem CID 52924278	Glycerophospholipids [GP]	MSI 2	18	15.59 (15.43 - 15.9)	15.25 (15.02 - 15.55)	15.62 (15.35 - 16.02)
PE(35:2)	729.5308	5.331017	PubChem CID 52924302	Glycerophospholipids [GP]	MSI 2	1	17.91 (17.07 - 18.15)	17.17 (16.8 - 17.55)	17.41 (17.11 - 17.64)
PE(35:3)	727.52	4.8389916	PubChem CID 52924362	Glycerophospholipids [GP]	MSI 2	8	12.55 (12.18 - 12.79)	12 (11.75 - 12.29)	12.24 (12.08 - 12.6)
PE(38:1)	773.5925	7.0100083	PubChem CID 52924721	Glycerophospholipids [GP]	MSI 2	21	14.75 (14.57 - 14.97)	14.42 (14.04 - 14.7)	14.76 (14.5 - 15.01)
PE(O-33:0)	691.5486	6.3850083	PubChem CID 52924882	Glycerophospholipids [GP]	MSI 2	13	12.19 (11.96 - 12.43)	11.88 (11.63 - 12.05)	12.03 (11.86 - 12.23)
PI(36:2)	858.5278	5.520012	PubChem CID 52928380	Glycerophospholipids [GP]	MSI 2	16	13.93 (13.51 - 14.18)	13.33 (13.12 - 13.67)	13.51 (13.23 - 13.97)

PS(40:0)	847.6329	7.2960043	PubChem CID 52925800	Glycerophospholipids [GP]	MSI 2	24	14.49 (14.44 - 14.68)	14.32 (14.2 - 14.42)	14.4 (14.23 - 14.52)
SM((43:2)	826.6846	7.655022	PubChem CID 52931223	Sphingolipids [SP]	MSI 2	20	15.18 (15.09 - 15.5)	14.98 (14.9 - 15.11)	15.1 (14.9 - 15.4)
DG(34:1)	616.5044	7.475991	Unknown	Glycerolipids [GL]	MSI 3	9	15.21 (14.85 - 15.58)	14.36 (13.9 - 15.22)	14.55 (13.9 - 15.13)
PC(P-38:3)	795.6136	7.2509837	Unknown	Glycerophospholipids [GP]	MSI 3	17	17.24 (17.12 - 17.38)	16.82 (16.62 - 16.99)	16.99 (16.89 - 17.23)
Unknown	598.4945	5.5109887	Unknown	Unknown	MSI 4	7	13.36 (12.98 - 13.89)	12.72 (12.44 - 13.07)	13.07 (12.67 - 13.47)
Unknown	804.0458	5.9179945	Unknown	Unknown	MSI 4	15	14.81 (14.54 - 14.83)	15 (14.92 - 15.08)	14.8 (14.63 - 14.93)
Unknown	808.0793	6.8029847	Unknown	Unknown	MSI 4	19	15.78 (15.6 - 15.84)	15.95 (15.84 - 16.04)	15.77 (15.68 - 15.88)
Unknown	743.5605	6.5889907	Unknown	Unknown	MSI 4	23	19.18 (19.11 - 19.5)	19.01 (18.89 - 19.21)	19.24 (19.04 - 19.39)
Unknown	1507.104	6.228019	Unknown	Unknown	MSI 4	10	14.65 (14.29 - 15.11)	14.23 (14.02 - 14.44)	14.62 (14.09 - 14.79)
Unknown	1549.144	6.250984	Unknown	Unknown	MSI 4	22	15.9 (15.62 - 16.02)	15.43 (15.15 - 15.66)	15.57 (15.26 - 15.78)
Unknown	508.3733	0.957998	Unknown	Unknown	MSI 4	14	12.01 (11.91 - 12.14)	11.71 (11.57 - 11.98)	11.85 (11.62 - 12.19)
Unknown	2392.67	5.9589906	Unknown	Unknown	MSI 4	12	13.92 (13.46 - 14.07)	14.22 (14.09 - 14.3)	14 (13.85 - 14.14)
Unknown	609.5346	7.097011	Unknown	Unknown	MSI 4	11	13 (12.8 - 13.27)	12.44 (11.99 - 12.86)	12.19 (11.8 - 12.86)

Supplementary table 2. G-mean, specificity and sensitivity for the machine learning algorithms trained and tested on the SR and CON samples.

Algorithm	G_mean	Specificity	Sensitivity
glmnet	0.7 (0.62 - 0.76)	0.6 (0.5 - 0.78)	0.78 (0.73 - 0.86)
knn	0.71 (0.64 - 0.77)	0.67 (0.55 - 0.82)	0.77 (0.68 - 0.83)
lda	0.55 (0.44 - 0.64)	0.5 (0.33 - 0.67)	0.64 (0.5 - 0.74)
pls	0.67 (0.56 - 0.74)	0.6 (0.42 - 0.75)	0.77 (0.7 - 0.86)
polr	0.49 (0.4 - 0.58)	0.45 (0.3 - 0.64)	0.57 (0.43 - 0.68)
rf	0.71 (0.63 - 0.76)	0.6 (0.45 - 0.78)	0.83 (0.74 - 0.91)
rpart	0.63 (0.53 - 0.69)	0.64 (0.44 - 0.78)	0.7 (0.55 - 0.83)
svmRadial	0.7 (0.58 - 0.78)	0.6 (0.4 - 0.78)	0.82 (0.74 - 0.91)
top4	0.72 (0.64 - 0.79)	0.67 (0.5 - 0.82)	0.82 (0.74 - 0.87)
